# COVID-19 breakthrough infections in type 1 diabetes mellitus: a cross-sectional study by the COVID-19 Vaccination in Autoimmune Diseases (COVAD) Group

**DOI:** 10.1007/s00296-023-05496-y

**Published:** 2023-12-07

**Authors:** Suhrud Panchawagh, Naveen Ravichandran, Bhupen Barman, Arvind Nune, Mahnoor Javaid, Abraham Edgar Gracia-Ramos, Jessica Day, Mrudula Joshi, Masataka Kuwana, Sreoshy Saha, Arunkumar R. Pande, Carlo Vinicio Caballero-Uribe, Tsvetelina Velikova, Ioannis Parodis, Johannes Knitza, Esha Kadam, Ai Lyn Tan, Samuel Katsuyuki Shinjo, Hiya Boro, COVAD Study Group, Parikshit Sen, Jessica Day, Elena Nikiphorou, Nelly Ziade, Marcin Milchert, Lorenzo Cavagna, Yi Ming Chen, Ashima Makol, Vishwesh Agarwal, Aarat Patel, John D. Pauling, Chris Wincup, Erick Adrian Zamora Tehozol, Jorge Rojas Serrano, Ignacia García-De La Torre, Iris J. Colunga-Pedraza, Javier Merayo-Chalico, Okwara Celestine Chibuzo, Wanruchada Katchamart, Phonpen Akawatcharangura Goo, Russka Shumnalieva, Leonardo Santos Hoff, Lina El Kibbi, Hussein Halabi, Binit Vaidya, Syahrul Sazliyana Shaharir, ATMTanveer Hasan, Dzifa Dey, Carlos Enrique Toro Gutiérrez, James B. Lilleker, Babur Salim, Tamer Gheita, Oliver Distler, Miguel A. Saavedra, Sinan Kardes, Laura Andreoli, Daniele Lini, Karen Schreiber, Melinda Nagy Vince, Yogesh Preet Singh, Rajiv Ranjan, Avinash Jain, Sapan C. Pandya, Rakesh Kumar Pilania, Aman Sharma, Manesh Manoj M, Vikas Gupta, Chengappa G. Kavadichanda, Pradeepta Sekhar Patro, Sajal Ajmani, Sanat Phatak, Rudra Prosad Goswami, Abhra Chandra Chowdhury, Ashish Jacob Mathew, Padnamabha Shenoy, Ajay Asranna, Keerthi Talari Bommakanti, Anuj Shukla, Arunkumar R. Pande, Kunal Chandwar, Akanksha Ghodke, Zoha Zahid Fazal, Döndü Üsküdar Cansu, Reşit Yıldırım, Armen Yuri Gasparyan, Nicoletta Del Papa, Gianluca Sambataro, Atzeni Fabiola, Marcello Govoni, Simone Parisi, Elena Bartoloni Bocci, Gian Domenico Sebastiani, Enrico Fusaro, Marco Sebastiani, Luca Quartuccio, Franco Franceschini, Pier Paolo Sainaghi, Giovanni Orsolini, Rossella De Angelis, Maria Giovanna Danielli, Vincenzo Venerito, Silvia Grignaschi, Alessandro Giollo, Alessia Alluno, Florenzo Ioannone, Marco Fornaro, Lisa S. Traboco, Suryo Anggoro Kusumo Wibowo, Jesús Loarce-Martos, Sergio Prieto-González, Raquel Aranega Gonzalez, Akira Yoshida, Ran Nakashima, Shinji Sato, Naoki Kimura, Yuko Kaneko, Takahisa Gono, Stylianos Tomaras, Fabian Nikolai Proft, Marie-Therese Holzer, Margarita Aleksandrovna Gromova, Or Aharonov, Zoltán Griger, Ihsane Hmamouchi, Imane El bouchti, Zineb Baba, Margherita Giannini, François Maurier, Julien Campagne, Alain Meyer, Daman Langguth, Vidya Limaye, Merrilee Needham, Nilesh Srivastav, Marie Hudson, Océane Landon-Cardinal, Wilmer Gerardo Rojas Zuleta, Álvaro Arbeláez, Javier Cajas, José António Pereira Silva, João Eurico Fonseca, Olena Zimba, Doskaliuk Bohdana, Uyi Ima-Edomwonyi, Ibukunoluwa Dedeke, Emorinken Airenakho, Nwankwo Henry Madu, Abubakar Yerima, Hakeem Olaosebikan, Becky A., Oruma Devi Koussougbo, Elisa Palalane, Ho So, Manuel Francisco Ugarte-Gil, Lyn Chinchay, José Proaño Bernaola, Victorio Pimentel, Hanan Mohammed Fathi, Reem Hamdy A. Mohammed, Ghita Harifi, Yurilís Fuentes-Silva, Karoll Cabriza, Jonathan Losanto, Nelly Colaman, Antonio Cachafeiro-Vilar, Generoso Guerra Bautista, Enrique Julio Giraldo Ho, Raúl González, Lilith Stange Nunez, Cristian Vergara M, Jossiell Then Báez, Hugo Alonzo, Carlos Benito Santiago Pastelin, Rodrigo García Salinas, Alejandro Quiñónez Obiols, Nilmo Chávez, Andrea Bran Ordóñez, Alberto Reyes Llerena, Radames Sierra-Zorita, Dina Arrieta, Eduardo Romero Hidalgo, Ricardo Saenz, Idania Escalante M, Wendy Calapaqui, Ivonne Quezada, Gabriela Arredondo, Rohit Aggarwal, Vikas Agarwal, Tulika Chatterjee, Latika Gupta

**Affiliations:** 1 Smt Kashibai Navale Medical College, Pune, India; 2 Department of Clinical Immunology and Rheumatology, Sanjay Gandhi Postgraduate Institute of Medical Sciences, Lucknow, India; 3 Department of Medicine, All India Institute of Medical Science (AIIMS), Guwahati, India; 4 Department of Rheumatology, Southport and Ormskirk Hospital NHS Trust, Southport, PR8 6PN UK; 5 Medical College, The Aga Khan University, Karachi, Pakistan; 6 Department of Internal Medicine, General Hospital, National Medical Center “La Raza”, Instituto Mexicano del Seguro Social, Av. Jacaranda S/N, Col. La Raza, C.P. 02990 Del. AzcapotzalcoMexico City, Mexico; 7 Department of Rheumatology, Royal Melbourne Hospital, Parkville, VIC 3050 Australia; 8 Walter and Eliza Hall Institute of Medical Research, Parkville, VIC 3052 Australia; 9 Department of Medical Biology, University of Melbourne, Parkville, VIC 3052 Australia; 10 Byramjee Jeejeebhoy Government Medical College and Sassoon General Hospitals, Pune, India; 11 Department of Allergy and Rheumatology, Nippon Medical School Graduate School of Medicine, 1-1-5 Sendagi, Bunkyo-ku, Tokyo, 113-8602 Japan; 12 Mymensingh Medical College, Mymensingh, Bangladesh; 13 LEDTC Clinic, Uttar Pradesh, Gomti Nagar, Lucknow, India; 14 Department of Medicine, Hospital Universidad del Norte, Barranquilla, Atlantico Colombia; 15 Medical Faculty, Sofia University St. Kliment Ohridski, 1 Kozyak Str., 1407 Sofia, Bulgaria; 16 Division of Rheumatology, Department of Medicine Solna, Karolinska Institutet and Karolinska University Hospital, Stockholm, Sweden; 17 Department of Rheumatology, Faculty of Medicine and Health, Örebro University, Örebro, Sweden; 18 Medizinische Klinik 3-Rheumatologie und Immunologie, Universitätsklinikum Erlangen, Friedrich-Alexander-Universität Erlangen-Nürnberg, Ulmenweg 18, 91054 Erlangen, Deutschland; 19 Seth Gordhandhas Sunderdas Medical College and King Edwards Memorial Hospital, Mumbai, Maharashtra India; 20 NIHR Leeds Biomedical Research Centre, Leeds Teaching Hospitals Trust, Leeds, UK; 21 Leeds Institute of Rheumatic and Musculoskeletal Medicine, University of Leeds, Leeds, UK; 22 Division of Rheumatology, Faculdade de Medicina FMUSP, Universidade de Sao Paulo, São Paulo, Brazil; 23 Department of Endocrinology and Metabolism, All India Institute of Medical Sciences, New Delhi, India; 24 Division of Rheumatology and Clinical Immunology, University of Pittsburgh School of Medicine, Pittsburgh, PA USA; 25 Center for Outcomes Research, Department of Internal Medicine, University of Illinois College of Medicine Peoria, Peoria, IL USA; 26 Department of Rheumatology, Royal Wolverhampton Hospitals NHS Trust, Wolverhampton, WV10 0QP UK; 27 Centre for Musculoskeletal Research, Division of Musculoskeletal and Dermatological Sciences, School of Biological Sciences, Faculty of Biology, Medicine and Health, Manchester Academic Health Science Centre, The University of Manchester, Manchester, UK; 28 Department of Rheumatology, City Hospital, Sandwell and West Birmingham Hospitals NHS Trust, Birmingham, UK

**Keywords:** COVID-19, Breakthrough infections, Type 1 diabetes mellitus, Vaccine, Survey

## Abstract

To investigate the frequency, profile, and severity of COVID-19 breakthrough infections (BI) in patients with type I diabetes mellitus (T1DM) compared to healthy controls (HC) after vaccination. The second COVID-19 Vaccination in Autoimmune Diseases (COVAD-2) survey is a multinational cross-sectional electronic survey which has collected data on patients suffering from various autoimmune diseases including T1DM. We performed a subgroup analysis on this cohort to investigate COVID-19 BI characteristics in patients with T1DM. Logistic regression with propensity score matching analysis was performed. A total of 9595 individuals were included in the analysis, with 100 patients having T1DM. Among the fully vaccinated cohort, 16 (16%) T1DM patients had one BI and 2 (2%) had two BIs. No morbidities or deaths were reported, except for one patient who required hospitalization with oxygen without admission to intensive care. The frequency, clinical features, and severity of BIs were not significantly different between T1DM patients and HCs after adjustment for confounding factors. Our study did not show any statistically significant differences in the frequency, symptoms, duration, or critical care requirements between T1DM and HCs after COVID-19 vaccination. Further research is needed to identify factors associated with inadequate vaccine response in patients with BIs, especially in patients with autoimmune diseases.

**Supplementary Information:**

The online version contains supplementary material available at 10.1007/s00296-023-05496-y.

## Introduction

The ongoing COVID-19 pandemic has significantly impacted individuals living with systemic autoimmune diseases (SAIDs) worldwide. A weaker immune response to infection along with the use of long-term immunosuppressive drugs put them at the risk of adverse clinical outcomes and mortality [1, 2]. Individuals with SAIDs such as T1 diabetes (T1DM), particularly those with poor glycaemic control are more susceptible to developing infections due to associated cytokine dysregulation and premature immunosenescence (accelerated aging of the immune system) in hyperglycemia [3]. Additionally, they may be particularly predisposed due to hyperglycemia and consequent immune dysregulation [4]. These patients may also have multiple complications of their disease (e.g. cardiovascular disease, chronic kidney disease) [5]. Hence, this patient subgroup is more likely to develop severe COVID-19 infection and its complications.

The development and widespread uptake of COVID-19 vaccines had been critical in containing and mitigating the impact of this virus. While current evidence suggests that the benefits of the vaccine outweigh its potential of vaccine-induced disease flares and other adverse outcomes [6, 7], the emergence of COVID-19 breakthrough infections (BI), defined as the detection of SARS-CoV-2 or antigen in the respiratory samples of an individual ≥ 14 days after complete vaccination, has raised concerns about the effectiveness of vaccines [8]. A diagnosis of systemic autoimmune diseases (SAIDs) is likely to render individuals at a higher risk of severe BI [9] contributing to vaccine hesitancy [10]. With limited evidence available on the safety of COVID-19 vaccines in T1DM patients, it is imperative to understand the prevalence and risk factors for BI in adults with T1DM.

This study aimed to investigate the frequency, profile, severity, and time to resolution of COVID-19 breakthrough infections (BI) in patients with type I diabetes mellitus (T1DM) compared to healthy controls (HC) after vaccination to elaborate the challenges and potential vulnerabilities faced by individuals with T1DM in the context of COVID-19 breakthrough infections, ultimately contributing to a more comprehensive understanding of vaccine efficacy and protection within this specific patient population.

## Methodology

### Study design

The second COVID-19 vaccination in autoimmune disease (COVAD-2), conducted in 2022, is a multinational, cross-sectional, patient-self-reported electronic survey [11]. This survey is a continuation of the first COVAD survey which was circulated in 2021 [9]. The first survey looked at short term vaccination-related adverse events, details of COVID-19 infection and health outcomes. The Checklist for Reporting Results of the Internet E-Surveys (CHERRIES) was adhered to when reporting the results [12]. The survey was administered to participants in over a 100 countries via > 150 centres. COVID-19 breakthrough infection (BI) data in patients with type 1 diabetes mellitus (T1D) and healthy controls (HC) was obtained.

### Data collection

After obtaining approval from worldwide experts, conducting a pilot test, and having the materials translated into 18 different languages, we hosted two extensive questionnaires (COVAD-1 AND COVAD-2) on the website surveymonkey.com. The survey was distributed by the COVAD study group internationally. Disease-specific information, such as the type of autoimmune rheumatic diseases (AIRD), duration, clinical symptoms, current medications, and patient-reported outcomes, such as PROMIS) physical and mental function and quality of life (QoL) scores, were also gathered in addition to patient demographics and comorbidities [13]. The specific type of COVID-19 vaccination administered to the patients included Pfizer-BioNTech, Oxford/Astra Zeneca, Johnson & Johnson (J&J), Moderna, Novavax, Covishield (Serum Institute of India), Covaxin (Bharat Biotech), Sputnik, Sinopharm, and Sinovac-CoronaVac, among others. We gathered information on breakthrough COVID infections in type 1 diabetics and healthy controls after vaccination. The previously published COVAD study protocol contain a full description of survey methodologies and questions [14].

### Data extraction

Following data collection, data were extracted from COVAD-2 database on 28th September 2022. Respondents excluded from the analysis are participants with incomplete responses, those who had their vaccinations in the middle of 2020 (likely trial participants), unvaccinated respondents, and those who had received only a single dose of any COVID-19 vaccine. We performed a subgroup analysis which included patients who had T1D from the cohort.

### Statistical analysis

Descriptive data are expressed as frequencies (percentages) and medians (inter-quartile ranges). Chi-squared (χ^2^) and Mann–Whitney U tests were used to compare groups for categorical and continuous variables, respectively. Propensity score matching analysis among BI in T1D and HCs was performed with 1:1 match and emphasis on exact matches. The factors matched were age, gender, ethnicity, presence of any comorbidity, and mental health disorders. Statistical analyses were performed using IBM SPSS version 28.

### Ethical considerations

Ethical approval was obtained from the Institutional Ethics Committee of SGPGIMS, Lucknow, India, and all participants consented electronically.

## Results

Of 10,783 respondents at the time of data analysis, 733 unvaccinated and 324 single-dose respondents, and 131 incomplete responses were excluded. Out of the total 9595 individuals included in the final analysis 3435 were HCs and 100 patients had the diagnosis of T1DM (Table S1 in the Supplementary File). Among the HCs, 467 (13.5%) reported experiencing a BI once and 124 (3.6%) reported experiencing a BI twice. Among the 100 T1DM patients (66% female, 46% Caucasian), 16 (16%) experienced one BI, and 2 (2%) experienced BI twice (Table S2 in the Supplementary File). The median time to symptom resolution was 10 days (2.5–72 days) in T1DM patients who received 2 doses of vaccine and 14 days (6 to 18 days) in those who received 3 doses. The most prevalent symptoms during the first BI in T1DM patients were cough (62.5%), fatigue (50%), fever (37.5%), and myalgia (31.25%). Only one patient required hospitalization for supplemental oxygen requirement, however, did not require intensive care.

Supplementary Table S3 presents the population characteristics of T1DM before and after PSM analysis. The results of PSM analysis indicated that individuals with T1DM had no statistically significant increase in the risk of experiencing one-time BI [T1DM vs HC OR 1.2 (0.7–2.0), p = 0.490] or two-time BI [T1DM vs HC OR 0.5 (0.1–2.2), p = 0.399] as compared to HCs (Table 1).

**Table 1:** Comparison of clinical features and management strategies between type 1 diabetic patients and healthy controls after propensity score matching

	T1DM (n = 14)	HC (n = 14)	OR (95% CI)	p value
Any symptoms	14 (100.0)	14 (100.0)		1.000
Fever	6 (42.9)	5 (35.7)		0.699
Fatigue	8 (57.1)	7 (50.0)		0.705
Muscle aches	5 (35.7)	9 (64.3)		0.131
Joint pains	3 (21.4	5 (35.7)		0.403
Cough	10 (71.4)	8 (57.1)		0.430
Difficulty breathing	2 (14.3)	5 (35.7)		0.190
Loss of smell	3 (21.4)	1 (7.1)		0.280
Loss of taste	3 (21.4)	2 (14.3)		0.622
Running nose	7 (50.0)	6 (42.9)		0.705
Congestion	5 (35.7)	7 (50.0)		0.445
Throat pain/scratchiness	5 (35.7)	9 (64.3)		0.131
Chest pain	0 (0)	1 (7.1)		0.309
Diarrhoea	5 (35.7)	4 (28.6)		0.686
Headache	4 (28.6)	9 (64.3)	0.2 (0.04–1.09)	0.058
Oral ulcers	2 (14.3)	3 (21.4)		0.622
Nausea/vomiting	0 (0)	0 (0)		–
Abdominal pain	1 (7.1)	3 (21.4)		0.280
Skin rashes	0 (0)	0 (0)		–
Hospitalisation	1 (7.1)	3 (21.4)		0.280
ICU care or other HDU	0 (0)	0 (0)		–
Oxygen requirement	0 (0)	1 (7.1)		0.309
Advanced treatment for COVID-19 infection	6 (42.9)	4 (28.6)		0.430

*T1DM* type 1 diabetes mellitus, *HC* healthy controls, *OR* odds ratio, *CI* confidence intervals, *ICU* intensive care unit, *HDU* high-dependency unit

## Discussion

In this study, we found no differences in the frequency, symptoms, duration, or critical care requirements between T1DM and HCs after COVID-19 vaccination. Individuals with SAIDs, including T1DM, may be at an increased risk of severe COVID-19 infection and associated morbidity and mortality when compared with healthy controls [15, 16]. This can be related to diabetes-associated hyperglycemia impairing the immune function and inducing immunosenescence [17, 18]. Moreover, hyperglycemia promotes viral replication via the production of mitochondrial reactive oxygen species and activation of hypoxia-inducible factor 1α [19], putting these patients at risk for BI even after full vaccination. A study by Basso et al. reported 2.41 times higher odds of contracting a BI in diabetics as compared to individuals without diabetes [20]. This contrasts with the reassuring findings of our study where there were no differences in the frequency of BIs between patients with T1DM and HCs. A recent study by Jia et al. reported similar humoral antibody responses to SARS-CoV-2 mRNA vaccines during 12 months of follow-up as well as similar rates of BI in T1DM patients and HCs [21].

Our study reported only mild symptoms following BI in T1DM patients including cough, fatigue, fever, and myalgia with only one patient requiring hospitalization for oxygen supplementation. The PRO-VACS 2 study reported mild COVID-19 symptoms including cough, cold, sore throat, and fever in cases of BI in 24 fully immunized T1DM patients [22]. The above study also did not show any major effect of BI on glycaemic control in vaccinated patients. Though some studies suggest a glycemic deterioration in diabetic patients following a COVID-19 infection [23], there is limited evidence regarding the effect of BI on glycemic control in these patients. In addition, a meta-analysis reported small improvements in various outcomes of glycemic control in diabetic patients with no statistically significant effect on HBA1c [24].

Our study did not find significant differences in BI frequency, clinical features, or severity between T1DM patients and HCs, reaffirming comparable protection provided by vaccinations in both groups [9, 25]. With limited evidence of vaccine effectiveness in T1DM available in literature, this brief report attempts to fill this gap and may serve as a valuable resource for clinicians when addressing patient inquiries regarding the frequency, severity, and time to resolution of BIs following COVID-19 vaccination. In addition, it can serve as a foundation for researchers to expand on the limited literature available on the impact of BIs on the long-term glycemic control of these patients, and the occurrence of any severe, life-threatening symptoms that may result from BIs.

COVAD group leads a large collaborative initiative with patient voice on COVID-19 infection and vaccination with data collected from over hundred countries, encompassing patient responses from a wide range of geographical areas. This is a major strength of our study. However, our study also has some limitations that should be considered. The sample size of T1DM patients is relatively small, data on glycaemic control was not captured by the survey, and there is the possibility of inadvertent introduction of recall and selection bias in the online survey. This anonymized, self-reported patient survey addresses the difference in perspective between patients and physicians about symptom burden and vaccine side effects. Understanding the patient’s perspective is imperative in addressing vaccine hesitancy. Further studies with larger sample sizes and longitudinal design may provide more definitive conclusions.

In conclusion, the results of our study suggest that vaccination against COVID-19 is as effective and safe in T1DM patients as compared to healthy controls. Further research to understand the factors associated with inadequate vaccine response in individuals with BIs will contribute to a more comprehensive understanding of BIs in the context of T1DM and autoimmune diseases.

### Supplementary Information

Below is the link to the electronic supplementary material.

Supplementary file1 (DOCX 74 KB)

## Data Availability

The datasets generated and/or analysed during the current study are not publicly available but are available from the corresponding author upon reasonable request.

## Declarations

### Disclaimer

No part of this manuscript has been copied or published elsewhere either in whole or in part.
