# Supplementary material for: COVID-19 breakthrough infections in type 1 diabetes mellitus: a cross-sectional study by the COVID-19 Vaccination in Autoimmune Diseases (COVAD) Group
Source: Rheumatol Int. 2023 Dec 7;44(1):73–80. doi: 10.1007/s00296-023-05496-y (PMC10766674; doi:10.1007/s00296-023-05496-y)
Supplement: Supplementary file 1 — Supplementary file1 (DOCX 74 KB) [file 296_2023_5496_MOESM1_ESM.docx]

**Supplementary Material**

Supplementary tables

Table S1: Baseline patient characteristics grouped by type 1 diabetes mellitus (overall and those with COVID-19 breakthrough infections

|  | T1D Total (n=100) | T1D with BI once (n=16) | HC total (n=3435) | HC with BI once (n=467) |
| --- | --- | --- | --- | --- |
| Age (median, IQR) years | 51 (40-63) | 51 (41-66.5) | 39 (30-49) | 38 (31-48) |
| **Gender n (%)***  Male  Female | 31 (31.0)  66 (66.0) | 0 (0)  15 (93.8) | 1296 (37.7)  2082 (60.6) | 162 (34.7)  298 (68.3) |
| **Ethnicity n (%)***  African  Asian  Caucasian  Hispanic  Mixed  Native American  Others | 6 (6.0)  17 (17.0)  46 (46.0)  13 (13.0)  7 (7.0)  1 (1.0)  5 (5.0) | 2 (12.5)  2 (12.5)  8 (50.0)  0 (0)  2 (12.5)  0 (0)  0 (0) | 119 (3.5)  864 (25.2)  1005 (29.3)  877 (25.5)  237 (6.9)  29 (0.8)  124 (3.6) | 7 (1.5)  78 (16.7)  207 (44.3)  115 (24.6)  16 (3.4)  2 (0.4)  24 (19.4) |
| **Number of vaccine doses n (%)**  2  3  4 | 27 (27.0)  61 (61.0)  12 (12.0) | 5 (5.0)  9 (9.0)  2 (2.0) | 1180 (34.4)  1990 (57.9)  265 (7.7) | 181 (38.8)  235 (50.3)  51 (10.9) |
| **Number of COVID-19 infections n (%)**  1  2  3 or more | 31 (31.0)  3 (3.0)  1 (1.0) | 14 (87.5)  2 (12.5)  0 (0) | 1106 (32.2)  190 (5.5)  26 (0.8) | 447 (95.7)  16 (3.4)  4 (0.9) |
| COVID-19 antibody present in n (%) | 5/8 (62.5) | 3/5 (60.0) | 227/297 (76.4) | 59/91 (64.8) |
| AID multimorbidity n (%) | 69 (69.0) | 16 (100.0) | - | - |
| Mental health disorders n (%) | 31 (31.0) | 8 (50.0) | 627 (18.3) | 85 (18.2) |
| **PROMIS global (median, IQR)**  Physical health scores  Mental health scores  Pain VAS  Fatigue VAS  PROMIS SF10a  Fatigue 4a | 13 (11-14)  13.5 (11-15.7)  3 (0.2-5)  3 (4-4)  43 (36-48)  10 (8-12) | 13.5 (11-14.7)  11.5 (10-15)  4.5 (3-5)  3 (3-4)  38 (31-44.7)  11 (9-12.7) | 13 (12-15)  15 (13-17)  0 (0-2)  4 (3-5)  49 (45-50)  7 (4-7) | 13 (12-14)  15 (12-17)  0 (0-2)  4 (3-5)  49 (45-50)  8 (5-11) |
| T1D: type 1 diabetes mellitus, HC: healthy controls, BI: COVID-19 breakthrough infections, IQR: interquartile range, AID: autoimmune disease, PROMIS: Patient-Reported Outcomes Measurement Information System, VAS: visual analogue scale  * The remaining did not wish to disclose | | | | |

Table S2: Characteristics of first BI among T1D patients

|  | After 2 doses (n=5) | After 3 doses (n=9) | After 4 doses (n=2) | p-value* |
| --- | --- | --- | --- | --- |
| Symptom resolution time (median, IQR) days | 10 (2.5-72) | 14 (6-18) | - | 0.317 |
| Any symptoms | 4 | 9 | 2 | 0.309 |
| Fever | 3 | 3 | 0 | 0.309 |
| Fatigue | 3 | 5 | 0 | 0.315 |
| Muscle aches | 2 | 3 | 0 | 0.575 |
| Joint pains | 2 | 1 | 0 | 0.318 |
| Cough | 3 | 7 | 0 | 0.120 |
| Difficulty breathing | 1 | 1 | 0 | 0.756 |
| Loss of smell | 1 | 2 | 0 | 0.754 |
| Loss of taste | 1 | 2 | 0 | 0.764 |
| Running nose | 1 | 6 | 0 | 0.099 |
| Congestion | 1 | 4 | 0 | 0.380 |
| Throat pain/scratchiness | 1 | 4 | 0 | 0.380 |
| Chest pain | 0 | 0 | 0 | - |
| Diarrhoea | 2 | 3 | 0 | 0.575 |
| Headache | 0 | 4 | 0 | 0.126 |
| Oral ulcers | 0 | 0 | 0 | - |
| Nausea/vomiting | 1 | 1 | 0 | 0.756 |
| Abdominal pain | 1 | 0 | 0 | 0.309 |
| Skin rashes | 0 | 0 | 0 | - |
| Hospitalisation | 1 | 2 | 0 | 0.764 |
| ICU care or other HDU | 0 | 0 | 0 | - |
| Oxygen requirement | 0 | 1 | 0 | 0.660 |
| Advanced treatment for COVID-19 infection | 1 | 4 | 2 | 0.156 |
| *Mann Whitney U test for continuous variables and Kruskal Wallis test for categorical variables | | | | |

Table S3: Baseline variables in type 1 diabetics and healthy controls before and after propensity score matching

|  | Unmatched | | | Matched* | | |
| --- | --- | --- | --- | --- | --- | --- |
|  | T1DM with BI (n=16) | HC with BI (n=467) | p-value | T1DM with BI (n=14) | HC with BI (n=14) | p-value |
| Age (median, IQR) years | 51 (41-66.5) | 38 (31-48) | 0.001 | 51 (41-66.5) | 53.5 (41.7-62) | 0.804 |
| Gender  Male  Female | 0 (0)  15 (93.8) | 162 (34.7)  298 (68.3) | 0.008 | 0 (0)  14 (100.0) | 0 (0)  14 (100.0) | 1.000 |
| **Ethnicity**  African  Asian  Caucasian  Hispanic  Mixed  Native American  Others | 2 (12.5)  2 (12.5)  8 (50.0)  0 (0)  2 (12.5)  0 (0)  0 (0) | 7 (1.5)  78 (16.7)  207 (44.3)  115 (24.6)  16 (3.4)  2 (0.4)  24 (19.4) | 0.002 | 2 (14.3)  2 (14.3)  7 (50.0)  0 (0)  0 (0)  2 (14.3) | 1 (7.1)  2 (14.3)  9 (64.3)  2 (14.3)  0 (0)  0 (0) | 0.359 |
| Number of vaccine doses  2  3  4 | 5 (5.0)  9 (9.0)  2 (2.0) | 181 (38.8)  235 (50.3)  51 (10.9) | 0.831 | 4 (28.6)  9 (64.3)  1 (7.1) | 6 (42.9)  5 (35.7)  3 (21.4) | 0.280 |
| Number of COVID-19 infections  1  2  3 or more | 14 (87.5)  2 (12.5)  0 (0) | 447 (95.7)  16 (3.4)  4 (0.9) | <0.001 | 13 (92.9)  0 (0)  1 (7.1) | 13 (92.9)  1 (7.1)  0 (0) | 0.368 |
| Any comorbidity | 16 (100.0) | 114 (24.4) | <0.001 | 14 (100.0) | 14 (100.0) | 1.000 |
| Mental health disorders | 8 (50.0) | 85 (18.2) | 0.002 | 7 (50.0) | 8 (57.1) | 0.705 |
| COVID-19 antibody status | 3/5 | 59/91 | 0.724 | 3/4 | 3/3 | 0.541 |
| * Matched for age, gender, ethnicity, any comorbidity, mental health disorder by PSM  T1D: type 1 diabetes mellitus, BI: COVID-19 breakthrough infections, HC: healthy controls, IQR: interquartile range | | | | | | |

# COVID-19 Vaccination in Autoimmune Diseases-2 (COVAD-2) Study Group- Complete Author List and Affiliations

United Kingdom

1. **Dr Hector Chinoy**- 1. Division of Musculoskeletal and Dermatological Sciences, Centre for Musculoskeletal Research, School of Biological Sciences, The University of Manchester, Manchester, UK. 2. National Institute for Health Research Manchester Biomedical Research Centre, Manchester University NHS Foundation Trust, The University of Manchester, Manchester, UK. 3. Department of Rheumatology, Salford Royal NHS Foundation Trust, Manchester Academic Health Science Centre, Salford, UK
2. **Dr Elena Nikiphorou**- 1. Centre for Rheumatic Diseases, King’s College London, London, UK. 2. Rheumatology Department, King's College Hospital, London, UK
3. **Dr James B Lilleker**- Centre for Musculoskeletal Research, Division of Musculoskeletal and Dermatological Sciences, School of Biological Sciences, Faculty of Biology, Medicine and Health, Manchester Academic Health Science Centre, The University of Manchester, Manchester, UK; Manchester Centre for Clinical Neurosciences, Salford Royal NHS Foundation Trust, Salford, UK
4. **Dr John D Pauling**- 1. Bristol Medical School Translational Health Sciences, University of Bristol, UK. 2. Department of Rheumatology, North Bristol NHS Trust, Bristol, UK
5. **Dr Chris Wincup**- Department of Rheumatology, Division of Medicine, Rayne Institute, University College London, 5 University Street, London WC1E 6JF, UK; Centre for Adolescent Rheumatology Versus Arthritis at UCL, UCLH, GOSH, London, UK.
6. **Dr Armen Yuri Gasparyan-** Departments of Rheumatology and Research and Development, Dudley Group NHS Foundation Trust, Russells Hall Hospital, North Block, Clinical Research Unit, Dudley, West Midlands, DY1 2HQ, United Kingdom.

India

1. **Mr** **Parikshit Sen**- Maulana Azad Medical College, New Delhi, Delhi, India
2. **Dr Vishwesh Agarwal**- Mahatma Gandhi Mission Medical College, Navi Mumbai, Maharashtra, India.
3. **Dr Yogesh Preet Singh**- Division of Rheumatology and Clinical Immunology, Department of General Medicine, Himalayan Institute of Medical sciences, Swami Rama University, Jolly Grant, Dehradun - 248140, Uttarakhand, India
4. **Dr Rajiv Ranjan** -Clinical Immunology & Rheumatology at Columbia Asia, Palam Vihar, Gurgaon, Haryana, India
5. **Dr Avinash Jain**- Department of Clinical Immunology and Rheumatology, SMS Medical College and Hospital, Jaipur, Rajasthan
6. **Dr Sapan C Pandya**- Clinical Immunologist and Rheumatologist, Rheumatic Disease Clinic, Vedanta Institute of Medical Sciences, Navrangpura, Ahmedabad 380009, Gujarat.
7. **Dr Rakesh Kumar Pilania**- Pediatric Allergy Immunology Unit, Department of Pediatrics, Post Graduate Institute of Medical Education and Research, Chandigarh.
8. **Dr Aman Sharma**- Clinical Immunology and Rheumatology Services, Department of Internal Medicine, Post Graduate Institute of Medical Education and Research, Chandigarh.
9. **Dr Manesh Manoj M**- Department of Clinical Immunology and Rheumatology, AKG Memorial Hospital and Dr Shenoy’s CARE (Centre for Arthritis and Rheumatism Excellence), Kannur, Kerala
10. **Dr Vikas Gupta**- Rheumatology, Dayanand Medical College and Hospital, Ludhiana, Punjab 141001, India.
11. **Dr Chengappa G Kavadichanda**- Department of Clinical Immunology, Jawaharlal Institute of Postgraduate Medical Education and Research, Puducherry, India
12. **Dr Pradeepta Sekhar Patro**- Department of Clinical Immunology and Rheumatology, Sunshine Hospitals, Plot No 208, Cuttack Puri Road, Laxmisagar, Bhubaneshwar, Odisha.
13. **Dr Sajal Ajmani**- Arthritis and Rheumatology clinic, New Delhi, Delhi.
14. **Dr Sanat Phatak**- Department of Rheumatology and Immunology, KEM Hospital, Pune, Maharashtra
15. **Dr Rudra Prosad Goswami**- Department of Rheumatology, All India Institute of Medical Sciences, New Delhi, Delhi
16. **Dr Abhra Chandra Chowdhury**- Rheumatologist, AMRI Hospital, Dhakuria, Kolkata, West Bengal
17. **Dr Ashish Jacob Mathew**- Department of Clinical Immunology & Rheumatology, Christian Medical College and Hospital, Vellore, Tamil Nadu 632004
18. **Dr Padnamabha Shenoy**, Medical Director (CARE), Dr Shenoy’s CARE (Centre for Arthritis and Rheumatism Excellence), Kannur, Kerala
19. **Dr Ajay Asranna**, Department of Neurology, NIMHANS, Bengaluru, Karnataka
20. **Dr Keerthi Talari Bommakanti**, Consultant Rheumatologist, Yashoda hospital, Behind Hari Hara Kala Bhavan, Secunderabad - 500003, T.S. Hyderabad, Telangana
21. **Dr Anuj Shukla**- Niruj Rheumatology Clinic, 209 Rajvi Complex, Rambaug, Ahmedabad, 380008, Gujarat
22. **Prithvi Sanjeevkumar Gaur-** Smt. Kashibai Navale Medical and General Hospital, Pune, India
23. **Dr Mahabaleshwar Mamadapur-** Department of Clinical Immunology and Rheumatology, Sanjay Gandhi Postgraduate Institute of Medical Sciences, Lucknow, India
24. **Akanksha Ghodke**- Mahatma Gandhi Mission Medical College, Navi Mumbai, Maharashtra, India
25. **Dr Kunal Chandwar**- Department of Clinical Immunology and Rheumatology, King George's Medical University, Lucknow, Uttar Pradesh, India

Pakistan

- - - 1. **Dr Babur Salim**- Rheumatology Department, Fauji Foundation Hospital, Rawalpindi, Pakistan
      2. **Zoha Zahid Fazal.** Medical College, The Aga Khan University, Karachi, Pakistan
      3. **Mahnoor Javaid.** Medical College, The Aga Khan University, Karachi, Pakistan

Turkey

1. **Dr Sinan Kardeş**- Department of Medical Ecology and Hydroclimatology, Istanbul Faculty of Medicine, Istanbul University, Capa-Fatih, 34093, Istanbul, Turkey
2. **Dr Döndü Üsküdar Cansu**, Division of Rheumatology, Department of Internal Medicine, Eskişehir Osmangazi University, 26480, Eskişehir, Turkey
3. **Dr Reşit Yıldırım**- Division of Rheumatology. Osmangazi University, Turkey.

United States of America

- - - 1. **Dr Ashima Makol**- Division of Rheumatology, Mayo Clinic, Rochester, MN, USA
      2. **Dr Aarat Patel**: Bon Secours Rheumatology Center and Division of Pediatric Rheumatology, Department of Pediatrics, University of Virginia School of Medicine , Charlottesville, VA, USA

France

1. **Dr. Margherita Giannini**- Explorations fonctionnelles musculaires, service de physiologie, Hôpitaux universitaires de Strasbourg; EA3072, fédération de médecine translationnelle. France
2. **Dr François Maurier-** Service de Médecine Interne, Hôspital Robert Schuman, Rue de Champ Montoy, 57070 Vantoux, France
3. **Dr Julien Campagne-** Service de Médecine Interne, Hôspital Robert Schuman, Rue de Champ Montoy, 57070 Vantoux, France
4. **Dr Alain Meyer-** 1. Centre National de Référence des Maladies Systémiques et Auto-immunes Rares Grand-Est Sud-Ouest (RESO), Service de humatologie, Service de physiologie, Unité d’explorations fonctionnelles musculaires, Hôpitaux Universitaires de Strasbourg, Strasbourg, France; 2. EA3072, Fédération de Médecine Translationelle, Université de Strasbourg, Strasbourg, France. alain.meyer1@chru-strasbourg.fr

Italy

1. **Dr. Lorenzo Cavagna**- Rheumatology Unit, Dipartimento di Medicine Interna e Terapia Medica, Università degli studi di Pavia, Pavia, Lombardy, Italy
2. **Dr. Nicoletta Del Papa**- Unità operativa complessa (UOC) Day Hospital Reumatologia via Gaetano Pini 9, Centro Specialistico Ortopedico Traumatologico, Gaetano Pini-CTO, Milano, Italy
3. **Dr. Gianluca Sambataro**- Medico Immunologia e reumatologia presso, Artoreuma S.R.L., Cors S. Vito 53, 95030 Mascalucia, CT, Italy
4. **Dr. Atzeni Fabiola**- Rheumatology Unit,  University of Messina, Messina, Italy.
5. **Dr. Marcello Govoni**- Department of Medical Sciences, Complex Operative Unit and Rheumatology Unit of S.Anna University Hospital, University of Ferrara, Via A. Moro 8, 44124- Cona (FE), Italy
6. **Dr Simone Parisi**- Epidemiology Unit, Italian Society for Rheumatology, Milan, Italy; Rheumatology Unit, Azienda Ospedaliera Città della Salute e della Scienza di Torino, Torino, Italy.
7. **Dr Elena Bartoloni Bocci**- Department of Medicine and Surgery, MED/16- Rheumatology, Università degli studi di Perugia, P.zza Università - 06123 – Perugia, Italy.
8. **Dr. Gian Domenico Sebastiani**- U.O.C. Reumatologia, Ospedale San Camillo-Forlanini, Roma, Italy
9. **Dr Enrico Fusaro**- Rheumatology Unit, Azienda Ospedaliero-Universitaria Città della Salute e della Scienza di Torino, Torino, Italy
10. **Dr Marco Sebastiani**- Rheumatology Unit, University of Modena and Reggio Emilia, Azienda Ospedaliero-Universitaria Policlinico di Modena, Via del Pozzo, 41125, Modena, Italy.
11. **Dr Luca Quartuccio**- Clinic of Rheumatology, Department of Medicine (DAME), ASUFC, University of Udine, Udine, Italy
12. **Dr Franco Franceschini**- Rheumatology and Clinical Immunology Unit, Department of Clinical and Experimental Sciences, ASST Spedali Civili and University of Brescia, Italy.
13. **Dr Pier Paolo Sainaghi**- Department of Translational Medicine, Università del Piemonte Orientale UPO, Novara, Italy; Division of Internal Medicine, Immunorheumatology Unit, CAAD (Center for Translational Research on Autoimmune and Allergic Disease) Maggiore della Carità Hospital, Novara, Italy; IRCAD, Interdisciplinary Research Center of Autoimmune Diseases, Novara.
14. **Dr Giovanni Orsolini**- Department of Medicine, Rheumatology Unit, University of Verona, Verona, Italy.
15. **Dr Rossella De Angelis**- Rheumatology Unit, Department of Clinical and Molecular Sciences, Polytechnic University of Marche
16. **Dr Maria Giovanna Danielli**- Clinica Medica, Dipartimento di Scienze Cliniche e Molecolari, Università Politecnica delle Marche e Azienda Ospedali Riuniti, Ancona, Italy.
17. **Dr Vincenzo Venerito**- Department of Emergency and Organ Transplantations-Rheumatology Unit, University of Bari "Aldo Moro", Bari, Italy.
18. **Dr Silvia Grignaschi**- Rheumatology Unit, Dipartimento di Medicine Interna e Terapia Medica, Università degli studi di Pavia, Pavia, Lombardy, Italy
19. **Dr. Alessandro Giollo-** Division of Rheumatology, Department of Medicine, University of Padova Hospital Trust, Padova, Italy
20. **Dr Laura Andreoli**- 1. Rheumatology and Clinical Immunology Unit, ASST Spedali Civili and University of Brescia, 25123 Brescia, Italy. 2. Department of Clinical and Experimental Sciences, University of Brescia, 25123 Brescia, Italy.
21. **Dr Daniele Lini**- 1. Rheumatology and Clinical Immunology Unit, ASST Spedali Civili and University of Brescia, 25123 Brescia, Italy. 2. Department of Clinical and Experimental Sciences, University of Brescia, 25123 Brescia, Italy
22. **Dr Alessia Alunno**- Rheumatology Unit, University of Perugia, Perugia, Italy
23. **Dr Florenzo Iannone**- DETO-Department of Emergency and Organ Transplantation-Rheumatology Unit, University of Bari, Bari, Italy
24. **Dr Marco Fornaro**- DETO-Department of Emergency and Organ Transplantation-Rheumatology Unit, University of Bari, Bari, Italy

Philippines

1. **Dr Lisa S Traboco-** Department of Medicine, Section of Rheumatology, St. Luke's Medical Center-Global City, Taguig, Philippines.

Malaysia

- - - 1. **Dr. Syahrul Sazliyana Shaharir**- Faculty of Medicine, Universiti Kebangsaan Malaysia, 56000, Cheras, Kuala Lumpur, Malaysia

Indonesia

**Dr Suryo Anggoro Kusumo Wibowo**- Division of Rheumatology, Department of Internal Medicine, Faculty of Medicine, Universitas Indonesia/ Dr Cipto Mangunkusumo General Hospital, Jakarta, Indonesia

Mexico

1. **Dr Miguel A Saavedra**- Departamento de Reumatología Hospital de Especialidades Dr. Antonio Fraga Mouret, Centro Médico Nacional La Raza, IMSS, Mexico City, Mexico
2. **Dr Erick Adrian Zamora Tehozol**- Centro Médico Pensiones, Autoimmunity Division, Mérida, Yucatán, Mexico
3. **Dr Jorge Rojas Serrano**, Rheumatologist and clinical investigator, Interstitial Lung Disease and Rheumatology Unit, Instituto Nacional de Enfermedades Respiratorias, Mexico City, Mexico
4. **Dr Ignacio García-De La Torre-** Departamento de Inmunología y Reumatología, Hospital General de Occidente and University of Guadalajara, Guadalajara, Jalisco, Mexico
5. **Dr. Iris J. Colunga‑Pedraza**- Rheumatology Service, Facultad de Medicina y Hospital Universitario “Dr. JoseE. Gonzalez”, Universidad Autonoma de Nuevo Leon, Av. Francisco I. Madero yGonzalitos S/N, Colonia Mitras Centro, 64460 Monterrey, Nuevo Leon, Mexico
6. **Dr.** **Javier Merayo-Chalico**- Department of Immunology and Rheumatology, Instituto Nacional de Ciencias Médicas y Nutrición “Salvador Zubirán”, Mexico City, Mexico.

Spain

1. **Dr Raquel Aranega-** Systemic Autoimmune Diseases Unit, Vall d'Hebron General Hospital, Medicine Dept, Universitat Autónoma de Barcelona, Barcelona, Spain.
2. **Dr Jesús Loarce-Martos**- Rheumatology Department, Hospital Universitario Ramón y Cajal, Carretera de Colmenar Viejo, 9, 1 km, 28043, Madrid, Spain.
3. **Dr Sergio Prieto-González**- Department of Internal Medicine, Hospital Clinic of Barcelona, University of Barcelona, Barcelona, Spain. sprieto@clinic.cat

Brazil

1. **Dr Leonardo Santos Hoff**- School of Medicine, Universidade Potiguar (UnP), Brazil.

Japan

1. **Dr Akira Yoshida-** Department of Allergy and Rheumatology, Nippon Medical School Graduate School of Medicine, 1-1-5 Sendagi, Bunkyo-ku, Tokyo 113-8602, Japan
2. **Dr Ran Nakashima**- Department of Rheumatology and Clinical Immunology, Graduate School of Medicine, Kyoto University, 54 Shogoin-Kawahara-cho, Sakyo-ku, Kyoto 606-8507, Japan
3. **Dr Shinji Sato**- Division of Rheumatology, Department of Internal Medicine, Tokai University School of Medicine, 143 Shimokasuya, Isehara, 259-1193, Japan. shinsjam@tokai-u.jp
4. **Dr Naoki Kimura**- Department of Lifetime Clinical Immunology, Graduate School of Medical and Dental Sciences, Tokyo Medical and Dental University (TMDU), Tokyo, Japan. kimura.rheu@tmd.ac.jp
5. **Dr Yuko Kaneko**- Division of Rheumatology, Department of Internal Medicine, Keio University School of Medicine, Tokyo, Japan
6. **Takahisa Gono-** Department of Allergy and Rheumatology, Nippon Medical School Graduate School of Medicine, 1-1-5 Sendagi, Bunkyo-ku, Tokyo 113-8602, Japan.

Poland

1. **Dr Marcin Milchert-** Department of Internal Medicine, Rheumatology, Diabetology, Geriatrics and Clinical Immunology, Pomeranian Medical University in Szczecin, ul Unii Lubelskiej 1, 71-252, Szczecin, Poland

Switzerland

1. **Dr Oliver Distler**- Department of Rheumatology, University Hospital Zurich, University of Zurich, Zurich, Switzerland

Germany

1. **Dr Stylianos Tomaras**- Department of Rheumatology, Helios Clinic Vogelsang-Gommern, 39245 Gommern, Germany
2. **Dr Fabian Nikolai Proft**- Department of Gastroenterology, Infectiology and Rheumatology (including Nutrition Medicine), Charité - Universitätsmedizin Berlin, corporate member of Freie Universität Berlin and Humboldt- Universität zu Berlin, Berlin, Germany
3. **Dr Marie-Therese Holzer**: 1. Department of Pediatrics, Pediatric Rheumatology/Special Immunology, University Hospital Wuerzburg, Josef-Schneider-Str. 2, 97080, Wuerzburg, Germany. 2. Department of Internal Medicine III. (Nephrology and Rheumatology With Section Endocrinology), University Hospital Hamburg- Eppendorf, University Hospital Hamburg-Eppendorf, Martinistraße 52, 20246, Hamburg, Germany

Denmark

- - - 1. **Karen Schreiber**- 1. Danish Hospital for Rheumatic Diseases, 6400 Sønderborg, Denmark. 2. Department of Regional Health Research (IRS), University of Southern Denmark, 5230 Odense, Denmark. 3. Thrombosis and Haemostasis, Guys and St Thomas’ NHS Foundation Trust, London SE1 7EH, UK

Russian Federation

1. **Dr Margarita Aleksandrovna Gromova**, Pirogov Russian National Research Medical University (RNRMU), Moscow, Russian Federation

Israel

1. **Mr Or Aharonov**, Department of Gerontology, Faculty of Social Welfare and Health Science, University of Haifa, Haifa, Israel

Hungary

1. **Dr Melinda Nagy-Vincze**- 1. Division of Clinical Immunology, Faculty of Medicine, University of Debrecen, Móricz Zsigmond út 22, Debrecen, H-4032, Hungary. 2. Gyula Petrányi Doctoral School of Clinical Immunology and Allergology, University of Debrecen, Debrecen, Hungary
2. **Dr Zoltán Griger**: 1. Division of Clinical Immunology, Faculty of Medicine, University of Debrecen, Móricz Zsigmond út 22, Debrecen, H-4032, Hungary

Lebanon

1. **Dr Nelly Ziade**- 1. Rheumatology Department, Saint-Joseph University, Beirut, Lebanon. 2. Rheumatology Department, Hotel-Dieu de France Hospital, Beirut, Lebanon

Morocco

1. **Dr Ihsane Hmamouchi-** Professor (Associate), Laboratoire d'épidémiologie et de recherche clinique, La Faculté de Médecine et de Pharmacie de Rabat, Morocco
2. **Dr Pr Imane El bouchti**, Head of the Rheumatology Department, Mohammed VI University Hospital, Marrakech, Morocco
3. **Dr. Zineb Baba**: Department of Rheumatology, Mohammed VI University Hospital, Marrakech, Morocco

Ghana

1. **Dr Dzifa Dey**- Department of Medicine and Therapeutics, University of Ghana Medical School, College of Health Sciences, Korle-Bu, Accra, Ghana.

Nigeria

1. **Dr Uyi Ima-Edomwonyi-** of Internal Medicine, Lagos University Teaching Hospital, Lagos, Nigeria
2. **Dr Ibukunoluwa Dedeke**- Department of Medicine, University College Hospital Ibadan, Ibadan, Nigeria
3. **Dr Emorinken Airenakho**- Consultant Rheumatologist, Irrua Specialist Teaching Hospital, KM 87 Benin Auchi Rd, 310115, Irrua, Nigeria
4. **Dr Nwankwo Henry Madu**- Lecturer 1, Department of Medicine, Nnamdi Azikiwe University, Awka
5. **Dr Abubakar Yerima**- Department of Medicine, University of Maiduguri Teaching Hospital, Maiduguri, Borno State, Nigeria
6. **Dr Hakeem Olaosebikan**- Consultant Rheumatologist, Lagos State University Teaching Hospital/ Lagos State University College of Medicine,Ikeja, Lagos, Nigeria
7. **Dr Celestine Chibuzo Okwara** - Department of Medicine, University of Nigeria Teaching Hospital, Ituku-Ozalla/University of Nigeria, Enugu Campus, Enugu, Nigeria

Ethiopia

- - - 1. **Dr Becky A.-** Rheumatology Unit, Internal Medicine Department, Addis Ababa University, Addis Ababa ,Ethiopia

Mauritius

**Dr Ouma Devi Koussougbo**- Rheumatology, Victoria hospital, Mauritius.

Mozambique

**Dr Elisa Palalane**: Rheumatology and Internal Medicine, Hospital Central de Maputo, Maputo, Mozambique

Australia

1. **Dr Daman Langguth**- Department of Immunology, Sullivan Nicolaides Pathology, Brisbane, Queensland, Australia
2. **Dr Vidya Limaye**- Royal Adelaide Hospital, Associate Professor of Rheumatology, Discipline of Medicine, University of Adelaide, Australia
3. **Dr Merrilee Needham**- 1. Neurology Department, Fiona Stanley Hospital, Murdoch, Australia; 2. Institute for Immunology and Infectious Diseases, Murdoch University, Murdoch, Australia; 3. Perron Institute for Neurological and Translational Science, Nedlands, Australia; 4. University of Notre Dame, Fremantle, Australia.
4. **Dr Nilesh Srivastav**- Alfred Health, The Alfred, Caulfield Hospital, Sandringham Hospital, Melbourne, Victoria, Australia

Canada

1. **Dr Marie Hudson-** Department of Medicine, McGill University, Montreal, Quebec; Division of Rheumatology, Jewish General Hospital, Montreal, Quebec, and Lady Davis Institute, Jewish General Hospital, Montreal, Quebec, Canada
2. **Dr Océane Landon-Cardinal-** Department of Medicine, University of Montreal, Montreal, Canada. Department of Medicine, CHUM Research Centre, Montreal, Canada. Department of Internal Medicine and Clinical Immunology and Inflammation-Immunopathology-Biotherapy Department (I2B), Pitié-Salpêtrière University Hospital, AP-HP, East Paris Neuromuscular Diseases Reference Center, Inserm U974, Sorbonne Université, Paris, France; Center of Reference for Neuromuscular Disorders AOC, Department of Neurology, Bordeaux University Hospital, Bordeaux, France; Polyvalent and Oncologic Radiology Department, Musculoskeletal Unit, Pitié-Salpêtrière University Hospital, AP-HP, Paris, France.

Bulgaria

1. **Dr Russka Shumnalieva-** Clinic of Rheumatology, University Hospital "St. Ivan Rilski", Medical University-Sofia, Urvich Street 13, 1612, Sofia, Bulgaria

Colombia

- - - 1. **Dr. Carlos Enrique Toro Gutiérrez**- General Director, Reference Center for Osteoporosis, Rheumatology and Dermatology, Pontifica Universidad Javeriana Cali, Colombia.
      2. **Dr Wilmer Gerardo Rojas Zuleta**- Department of Rheumatology, Universidad de Antioquia, Cl. 67 #53 - 108, Medellín, Colombia
      3. **Dr. Álvaro Arbeláez- Médico especialista en Reumatología y Medicina Interna. Universidad Libre. Clínica Imbanaco. Clínica de Artritis Temprana. Cali, Valle, Colombia**
      4. **Dr. Javier Cajas-** Institute of Rheumatology Ferdinand Chalem, Bogota, Colombia

Portugal

**Dr José António Pereira Silva**- Rheumatology Department, Centro Hospitalar e Universitário de Coimbra EPE, and Coimbra Institute of Clinical and Biomedical Research (iCBR), Faculty of Medicine, University of Coimbra, Portugal

**Dr João Eurico Fonseca**- Hospital de Santa Maria, Centro Hospitalar Lisboa Norte Centro Académico de Medicina de Lisboa, Lisboa, Portugal; Instituto de Medicina Molecular, Faculdade de Medicina, Universidade de Lisboa, Lisboa, Portugal

Ukraine

**Dr Olena Zimba-** Department of Internal Medicine #2, Danylo Halytsky Lviv National Medical University, Lviv, Ukraine

**Dr Doskaliuk Bohdana**- Department of Pathophysiology, Ivano-Frankivsk National Medical University, Ivano-Frankivsk, Ukraine

Hong Kong

1. **Dr Ho So**- Assistant Professor, Department of Medicine & Therapeutics, Faculty of Medicine, The Chinese University of Hong Kong, Hong Kong

Peru

1. **Dr Manuel Francisco Ugarte-Gil**- Servicio de Reumatología, Hospital Nacional Guillermo Almenara Irigoyen, EsSalud, Lima, Peru. School of Medicine, Universidad Científica del Sur, Lima, Peru
2. **Dr. Lyn Chinchay**: Seguro Social de Salud del Peru (ESSALUD), Lima, Peru
3. **Dr. José Proaño Bernaola**: 1. Cayetano Heredia National Hospital, Lima, Peru. 2. Anglo-American Clinic. San Isidro, Lima, Peru. 3. Peruvian University Cayetano Heredia, Lima, Peru. 4. Rheumatologist, San Judas Tadeo Clinic - Research Center (ENDOMED), Lima. 5. Environmental and Food Health - DIRIS – Lima
4. **Dr. Victorio Pimentel**: Rheumatology Department, Hospital Guillermo Almenara Irigoyen, EsSalud, Av. Grau 800, La Victoria, Lima 13, Lima, Peru

Bangladesh

1. **Dr A.T.M. Tanveer Hasan**- Department of Rheumatology, Enam Medical College and Hospital, 9/3 Parboti Nagar, Thana Rd, Savar Union 1340, Bangladesh

Nepal

1. **Dr Binit Vaidya**- Department of Rheumatology, National Centre for Rheumatic Diseases, Ratopul, Kathmandu, Nepal

Egypt

1. **Dr Tamer A Gheita**- Rheumatology Department, Kasr Al Ainy School of Medicine, Cairo University, Cairo, Egypt
2. **Dr Hanan Mohamed Fathi**- Rheumatology and Autoimmune Diseases, Faculty of Medicine, Fayoum University, Faiyum, Egypt
3. **Dr Reem Hamdy A Mohammed**: Department of Rheumatology and Clinical Immunology, Kasr Alainy School of Medicine- Cairo University, Cairo, Egypt.

Taiwan

1. **Dr Yi-Ming Chen**- Division of Allergy, Immunology and Rheumatology, Department of Medical Research, Taichung Veterans General Hospital, Taiwan

United Arab Emirates

1. **Dr Ghita Harifi**- Department of Rheumatology, Mediclinic Parkview Hospital, 3 Umm Suqeim St - Al Barsha Al Barsha South, Dubai, United Arab Emirates.

Saudi Arabia

1. **Dr Lina El Kibbi**- Department of Rheumatology, Specialised Medical Center Hospital, Alfaisal University, Riyadh, Saudi Arabia
2. **Dr Hussein Halabi**- Department of internal medicine, Section of rheumatology, King Faisal Specialist Hospital and Research Center, Jeddah, Saudi Arabia

Thailand

- - - 1. **Dr Phonpen Akawatcharangura Goo**- Department of Medicine, Queen Savang Vadhana Memorial Hospital, Chonburi, Thailand.

1. **Dr. Wanruchada Katchamart-** Division of Rheumatology, Department of Medicine, Faculty of Medicine Siriraj Hospital, Mahidol University, Bangkok, Thailand.

Venezuela

1. **Dr. Yurilís Fuentes-Silva**: 1. Health Sciences School, University of Oriente– Bolivar Nucleus, Ciudad Bolivar, Venezuela. 2. Centro Clínico Universitario de Oriente, Ciudad Bolivar, Venezuela

Paraguay

1. **Dr. Karoll Cabriza:** Hospital de Clínicas, San Lorenzo, Paraguay
2. **Dr.** **Jonathan Losanto**: Hospital de Clínicas, San Lorenzo, Paraguay.
3. **Dr. Nelly Colaman**: Hospital de Clínicas, San Lorenzo, Paraguay.

Panama

1. Dr. **Antonio Cachafeiro-Vilar**: Pacífica Salud-Hospital Punta Pacífica, Ciudad de Panamá, Panamá
2. **Dr. Generoso Guerra Bautista**: Centro de Investigación Marbella, Paitilla Panamá, Panamá
3. **Dr. Enrique Julio Giraldo Ho**: Rheumatologist, Universidad de Panamá, República de Panamá

Chile

1. **Dr. Lilith Stange Nunez**: Rheumatologist, University of Valparaiso, Pontifical Catholic University of Chile
2. **Dr. Cristian Vergara M**: Departamento de Medicina Interna, Escuela de Medicina Dirección de PostGrado y Post Título, Facultad de Medicina, Universidad de Valparaíso, Santiago, Chile

Dominican Republic

1. **Dr. Jossiell Then Báez**: MD, Hospital Metropolitano de Santiago (HOMS), Santiago, Dominican Republic

Honduras

1. **Dr. Hugo Alonzo**- Jefe del Departamento de Medicina Interna en Hospital de Especialidades del Seguro Social en Tegucigalpa, Honduras
2. **Dr. Carlos Benito Santiago Pastelin**- Médico especialista, Instituto Hondureño del Seguro Social, Honduras

Argentina

1. **Dr. Rodrigo García Salinas**- Rheumatology Unit, La Plata Italian Hospital, Buenos Aires, Argentina

Guatemala

1. **Dr. Alejandro Quiñónez Obiols**- Universidad Mariano Gálvez de Guatemala, Guatemala City, Guatemala.
2. **Dr. Nilmo Chávez**- Instituto Guatemalteco de Seguridad Social - Universidad San Carlos de Guatemala, Ciudad de Guatemala, Guatemala
3. **Dr. Andrea Bran Ordóñez**- Hospital El Pilar, Universitario Esperanza, Guatemala City, Guatemala

Cuba

1. **Dr. Gil Alberto Reyes Llerena**- Surgical Medical Research Center (CIMEQ), Rheumatology Service, 216th Street and 11B, Siboney. Beach. Havana, Cuba

Puerto Rico

1. **Dr. Radames Sierra-Zorita**- University of Puerto Rico, School of Medicine, San Juan, Puerto Rico

Costa Rica

1. **Dr. Dina Arrieta**- Hospital México, Caja Costarricense del Seguro Social, San José de Costa Rica, Costa Rica
2. **Dr. Eduardo Romero Hidalgo**- Jackson's Memorial Medical Center, San Ramon, Alajuela, Costa Rica
3. **Dr. Ricardo Saenz**- Jefe Servicio Reumatología Hospital Dr. Calderón Guardia C.C.S.S.

Nicaragua

1. **Dr. Idania Escalante M**- 1. Internal Medicine, Rheumatologist, Oscar Danilo Rosales School Hospital. Leon-Nicaragua. 2. Medicine professor of National Autonomous University of Nicaragua, Nicaragua

Ecuador

1. **Dr. Wendy Calapaqui**- Instituto Ecuatoriano de Seguridad Social. Centro de atención ambulatoria “El Batán”, Quito, Ecuador
2. **Dr. Ivonne Quezada**- Hospital de Especialidades Eugenio Espejo, Quito, Ecuador

Bolivia

1. **Dr. Gabriela Arredondo**: Department of Rheumatology, Medical Center, Santa Cruz, Bolivia; Department of Rheumatology, Alemana Clinic, La Paz, Bolivia
